# Patellar maltracking: an update on the diagnosis and treatment strategies

**DOI:** 10.1186/s13244-019-0755-1

**Published:** 2019-06-14

**Authors:** Zaid Jibri, Paul Jamieson, Kawan S. Rakhra, Marcos L. Sampaio, Geoffrey Dervin

**Affiliations:** 1 Department of Medical Imaging, The Ottawa Hospital, 501 Smyth Road, Ottawa, ON K1H 8L6 Canada; 2 Faculty of Medicine, University of Ottawa, 451 Smyth Road, Ottawa, ON K1H 8M5 Canada; 3 Division of Orthopaedic Surgery, The Ottawa Hospital, 501 Smyth Road, Ottawa, ON K1H 8L6 Canada

**Keywords:** Patellar maltracking, Patellar instability, Anterior knee pain, Patellofemoral osteoarthritis

## Abstract

Patellar maltracking occurs as a result of an imbalance in the dynamic relationship between the patella and trochlea. This is often secondary to an underlying structural abnormality. The clinical evaluation can provide useful clues for the presence of such entity; however, the diagnosis can often be challenging especially in the absence of a documented history of patellar dislocation. Imaging, particularly MRI, can detect subtle features that could lead to the diagnosis, probably even more importantly when there is no clear history of patellar dislocation or before its development. This can provide a road map for formulating a treatment strategy that would be primarily aimed at stabilizing the patellofemoral joint to halt or slow the progression of articular cartilage loss. The purpose of this article is to discuss the clinical and radiologic evaluation of patellar maltracking providing an update on the cross-sectional imaging assessment and also a synopsis of the management options.

## Key points

- The clinical evaluation of patellar maltracking is often challenging
- Imaging can detect subtle features that could lead to early diagnosis
- Imaging can detect predisposing factors for patellar maltracking and associated structural changes
- Management decisions are made on individual basis with imaging playing a vital role

## Background

Patellar tracking refers to the dynamic relationship between the patella and trochlea during knee motion [1]. Patellar maltracking occurs as a result of imbalance of this relationship often secondary to anatomic morphologic abnormality. Usually, young individuals, particularly women, suffer the consequences of this disorder [2]. It is a recognized cause of anterior pain and in extreme cases presents as acute and often recurrent patellar dislocation, which is usually transient. Early diagnosis is essential, as chronic maltracking will lead to patellofemoral cartilage damage and osteoarthritis [3]. Imaging, particularly MRI, plays a vital role in the assessment of patellar maltracking. It can not only detect any underlying morphological risk factors but also look for structural damage associated with maltracking including patellofemoral articular cartilage loss, osteochondral defects, or damage to the medial patellar stabilizers [4, 5]. The purpose of this article is to discuss the evaluation of patellar maltracking providing an update on the imaging assessment and also a synopsis on the management options.

### Biomechanics of the patellofemoral joint

The knee is a complex joint with separate tibio-femoral and the patellofemoral articulations. Understanding the biomechanics of these joints is essential to investigating and appropriately treating patellofemoral joint pathology. The patellofemoral joint has two primary functions; firstly, it acts as an anatomic pulley to provide mechanical advantage for the extensor mechanism and, secondly, to reduce friction between the extensor mechanism and the femur. The patella itself is shaped as an inverted triangle and is embedded in the quadriceps tendon, making it the largest sesamoid bone in the body [6]. Distally, it attaches to the tibial tubercle via the patellar tendon. The posterior articulating surface of the patella is composed of two facets, a medial and lateral facet, separated by a vertical ridge, and in 30% of the population, there is a third facet, the odd facet, most medially. The patella articulates with the trochlear groove of the anterior femur, which has corresponding lateral and medial patellar articular surfaces [6]. The lateral trochlear articular surface is usually more prominent than its medial portion. As the knee joint ranges from extension to flexion, the articular surface area of the patella is in contact with the femur changes. In full extension, the patella has little to no contact with the trochlear groove and, therefore, is in a position of higher risk for instability. From 10 to 20° of flexion, the patella engages the trochlear groove with the contact area being the inferior most portion of the medial and lateral facets. As the knee progresses through greater flexion, the contact surface becomes more proximal on the patella. It is not until beyond 90° of flexion that the odd facet engages the medial femoral condyle and plays a role in load sharing along with lateral facet [6, 7]. Additionally, in this degree of flexion, the quadriceps tendon itself engages the proximal trochlear groove and participates in force distribution [8–10].

The patella has 4 different planes of motion: flexion–extension, medial–lateral rotation, medial–lateral patellar tilt, and medial–lateral patellar shift. The stability of the patella is dependent on both osseous anatomy and the integrity of longitudinal and transverse soft tissue stabilizers. The transverse stabilizers include the medial and lateral retinaculum, the vastus medialis and lateralis muscles, the ilio-tibial band, and the medial patellofemoral ligament (MPFL). The longitudinal stabilizer is the extensor mechanism itself, which is comprised of the quadriceps tendon proximally and the patellar tendon distally. The anatomic relationship between the resultant force from the quadriceps and the line of pull of the patellar tendon is termed the Q angle and is normally 10–15° of valgus [11]. This results in a slightly superolateral direction of pull on the patella by the quadriceps. Correspondingly, the patella must shift slightly medially during early flexion to engage the trochlear groove.

### Clinical presentation and initial evaluation

The most obvious presentation of patellar maltracking is that of the first time lateral patellar instability or recurrent instability thereafter. Less commonly, patients can also present after chronic patellar instability secondary to generalized ligamentous laxity with or without anterior knee pain. Although varied in presentation, successful management of all patients relies on thorough history taking, physical examination of the entire lower extremity, and appropriate imaging. The clinical evaluation can be more challenging in the absence of a dislocation history, and in this scenario, imaging can have a critical role.

Acute traumatic instability most commonly occurs in young athletes in their second and third decade at an incidence rate of 29 per 100,000. Some controversy exists regarding whether female gender is a definite risk factor for patellar instability with certain studies identifying a 33% increased likelihood of first-time dislocation as well as three times high re-dislocation rates than males, whereas others have found roughly equal rates [2, 12–14]. The mechanism is commonly a non-contact twisting injury of the lower extremity with the knee extended and external rotation of the foot and is perceived as the knee “giving way.” The patella will often self-reduce by reflexic contraction of the quadriceps muscles. Less commonly, a direct laterally or medially orientated blow to the patella can precipitate dislocation. Traumatic dislocations are commonly associated with other injuries including that of the MPFL, meniscal pathology, and osteochondral fractures of the femur or patella [15, 16].

Between 15 and 45% of patients will develop recurrent patellar instability after acute dislocation, which is both functionally limiting and painful [17–20]. Risk factors for recurrent instability include female sex, family history of patellar instability, and various anatomic risk factors such as patella alta, increased femoral anteversion, external tibial rotation, genu valgum, trochlear dysplasia, increased tibial tubercle–trochlear groove (TT-TG) distance, and patellar tilt [13, 21–23]. These prevailing anatomic indices feature prominently into the probability of recurrence, and understanding their variability and pathophysiology is critical to successful management of these patients.

A focused history of the mechanism, number, and circumstances of instability to date is essential. A generalized physical examination assessing ligamentous laxity and rotational profile of the lower extremity is critical. A thorough examination of the knee is then performed including presence of effusion, localization of pain, assessment of patellar translation, patellar apprehension, presence of a J sign (visual lateralization of the patella as it disengages from the trochlea when extending the knee), and a measurement of the Q angle along with ligamentous and meniscal testing. Distal neurovascular examination also needs to be performed [16].

### Imaging techniques

Imaging assessment can start with the radiograph including anteroposterior and lateral views of the knee and skyline view of the patella. The radiograph can be helpful in the acute presentation in detecting fractures in the setting of lateral (often transient) patellar dislocation. However, it lacks sensitivity with 40% of sizable osteochondral lesions being missed on initial presentation after patellar dislocation [16]. The radiograph can also be useful in detecting osseous morphologic features associated with patellar maltracking such as patella alta and trochlear dysplasia [24, 25]. MRI and CT are superior modalities in looking for predisposing factors associated with patellar maltracking [26–28]. In this section, we will emphasize the role of MRI and discuss how CT can also have value when assessing patellar maltracking.

### Magnetic resonance imaging features

Magnetic resonance imaging (MRI) is a vital tool in evaluating the potential cause(s) of anterior knee pain due to the complexity of the structure and biomechanics of the knee. Various parameters can be used in assessing and predicting the presence of patellar maltracking. These parameters can be evaluated using dynamic MRI [29]. However, the use of this method is not widespread. On the other hand, there are static MRI measurements that are routinely used as indicators of patellofemoral alignment during knee movement [30, 31]. The main morphological features associated with patellar maltracking are trochlear dysplasia, lateralization of the tibial tuberosity, patella alta, and lateral patellar tilt. MRI is the imaging modality of choice in the assessment of patellar maltracking, as a virtue of what it can reveal (Table 1).

**Table 1:** MRI checklist for the assessment of patellar maltracking

Patellar maltracking-associated feature	Methods of assessment (amongst others)	Significance
Trochlear dysplasia	Trochlear depth (Fig. 1), lateral trochlear inclination (Fig. 2), trochlear facet asymmetry (Fig. 3) (evaluated on most cranial axial image showing cartilage, approximately 3 cm above the joint line)	Geometric abnormality of the trochlear groove that can result in abnormal tracking of the patella along the trochlea
Patella alta	Insall–Salvati index (Fig. 4) Caton–Deschamps index (Fig. 4)	Relates to a long patellar tendon. In order for the patella to engage with the trochlea, a higher degree of flexion than normal is needed
Lateralization of the tibial tuberosity	Tibial tubercle–trochlear groove distance (TT-TG) (Fig. 5)	High TT-TG would exert lateral pressure on the patella during extension, and if not counteracted by vastus medialis contraction, it may predispose to patellar subluxation
Lateral patellar tilt	Patellar tilt angle (Fig. 6) Patellofemoral angle	Sensitive marker for patellar instability present in significant proportion of patients
Hoffa’s fat pad impingement	Edema at the superolateral aspect of Hoffa’s fat pad on MRI	Significant association with several patellar maltracking indicators
MPFL and medial patellar retinacular injury	Best evaluated on the axial fluid sensitive MRI sequence	Present in the majority of patellar dislocation cases
Chondral and osteochondral damage	MRI can show discrete osteochondral defect or various degrees of patellofemoral cartilage loss	Patellar maltracking is significant risk factor patellofemoral osteoarthritis. Patellar dislocation can result in discrete osteochondral defects at the patella or lateral femoral condyle

### Trochlear dysplasia

**Fig. 1:**
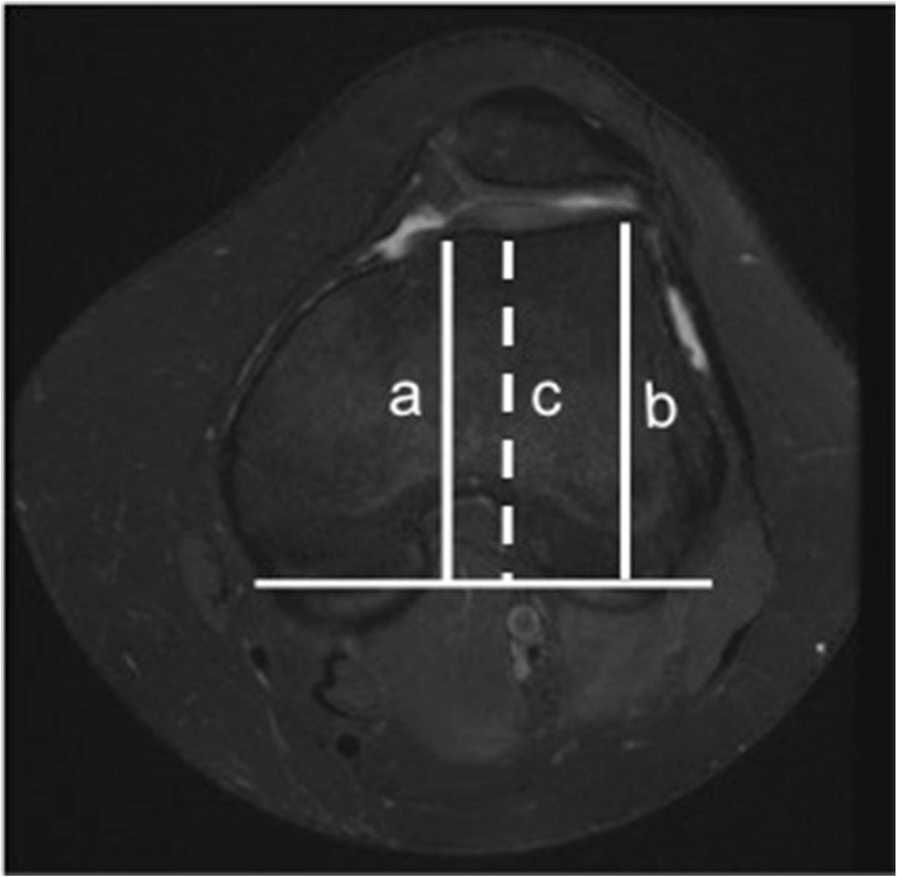
Trochlear depth assessment. Axial PDFS left knee MR image, demonstrating the method used for the measurement of trochlear depth. First, a line is drawn paralleling the posterior femoral condyles surfaces. Perpendicular to this baseline, trochlear depth is calculated by measuring the mean of the maximum AP distance of the medial (**a**) and lateral (**b**) femoral condyles minus the distance between the deepest point of the trochlear groove and the line paralleling the posterior condylar surfaces (**c**). Trochlear depth = [(a + b)/2] − c

**Fig. 2:**
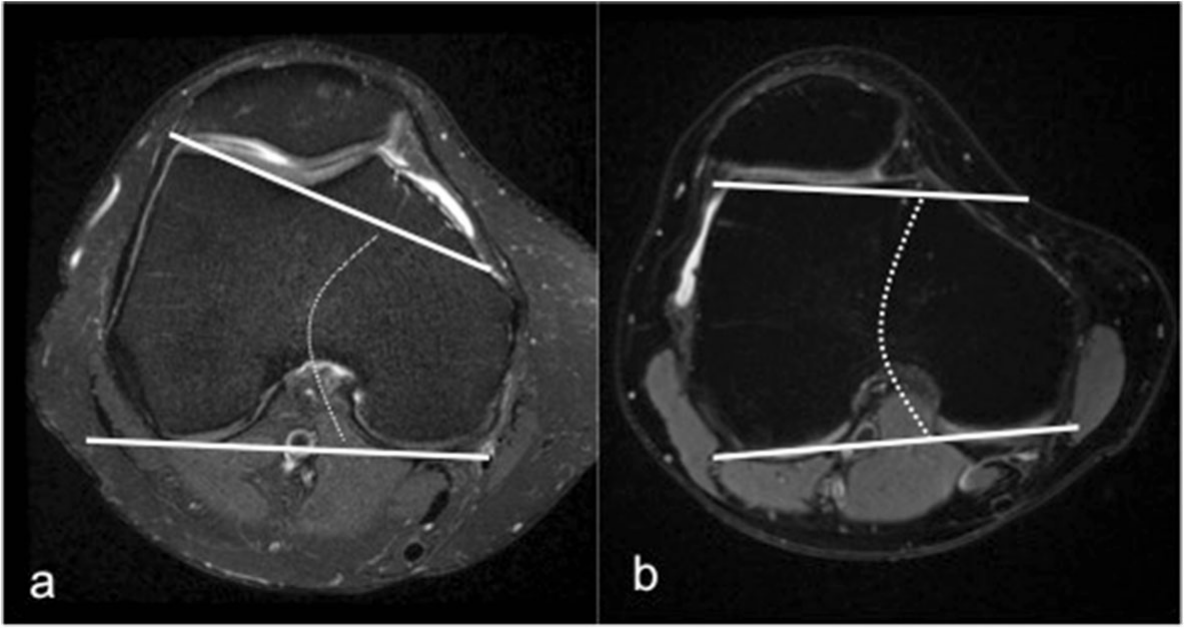
Lateral trochlear inclination measurement on axial MRI. It is the angle between a line tangential to the subchondral bone of the posterior aspect of the femoral condyles and a line along the lateral trochlear facet. **a** Normal trochlea. **b** Trochlear dysplasia (9° inclination)

**Fig. 3:**
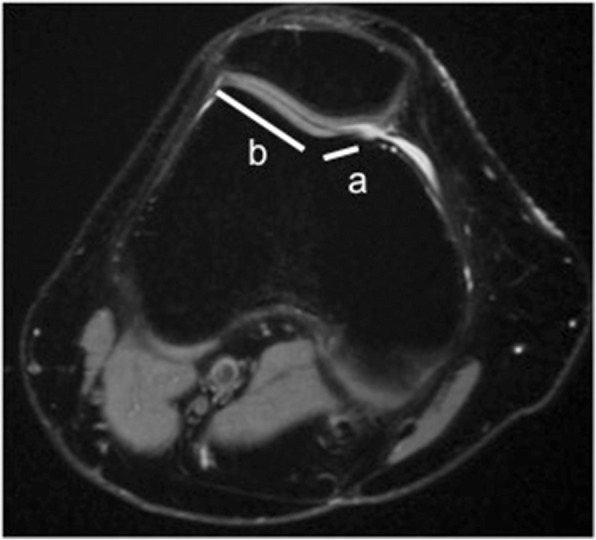
Facet asymmetry assessment for trochlear dysplasia on axial MRI. It is the percentage of the medial (**a**) to the lateral (**b**) trochlear facet length (a/b × 100%)

It is a geometric abnormality of the trochlear groove that affects its shape and depth mainly at its superior part, which can result in abnormal tracking of the patella along the trochlea. It is a major factor in patellar instability and was shown to be present in 85% of these patients [21]. There are a number of MRI features of trochlear dysplasia including reduction in the trochlear depth, lateral trochlear inclination, and facet asymmetry. These are evaluated on most cranial axial image showing cartilage, approximately 3 cm above the joint line. The trochlear depth is calculated by measuring the mean of the maximum anteroposterior (AP) distance of the medial and lateral femoral condyles minus the distance between the deepest point of the trochlear groove and the line paralleling the posterior femoral condyles surfaces (Fig. 1). Less than 3-mm trochlear depth is indicative of trochlear dysplasia [24]. Lateral trochlear inclination is another quantitative method to diagnose trochlear dysplasia. It is the angle between a line tangential to the subchondral bone of the posterior aspect of the femoral condyles and a line along the lateral trochlear facet subchondral bone (Fig. 2). An inclination angle of less than 11° indicates trochlear dysplasia [32]. Facet asymmetry is determined by calculating the percentage of the medial to the lateral femoral facet length (Fig. 3). Asymmetry of < 40% suggests trochlear dysplasia [24].

Dejour et al. provided a morphologic classification system for trochlear dysplasia describing four types [26–28]. In type A, the trochlear preserves its concave shape but has shallow trochlear groove; type B is flattened or convex trochlea; in type C, the medial facet is hypoplastic (facet asymmetry) with high lateral facet, resulting in flattened joint surface in an oblique plane; and type D shows a “cliff pattern” with type C features and a vertical link between the medial and lateral facets.

### Patella alta

**Fig. 4:**
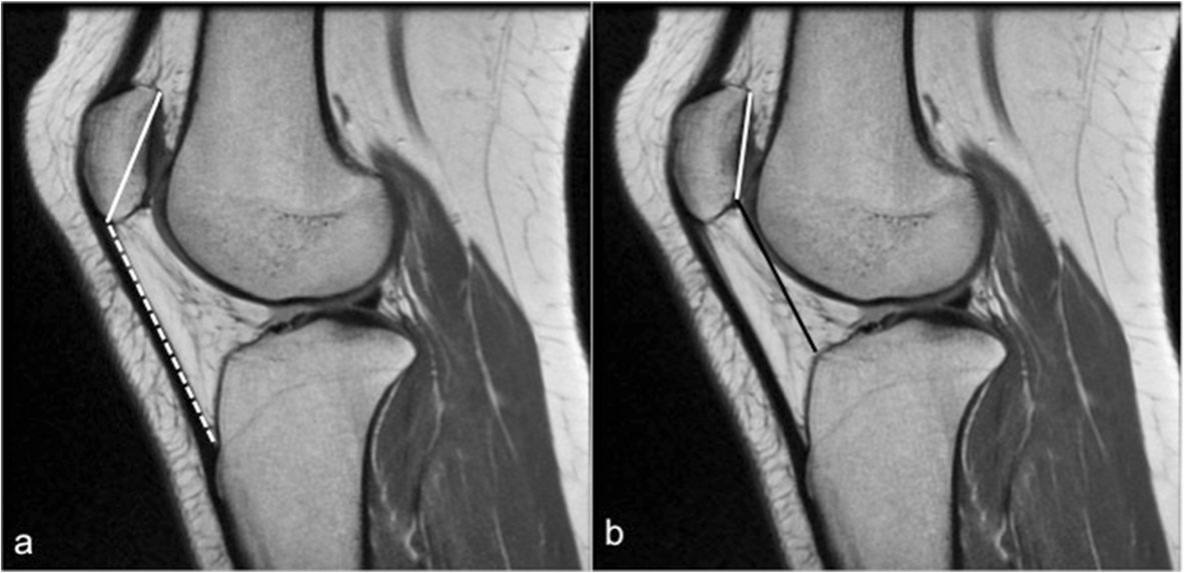
Patella alta assessment. **a** Sagittal PD knee MRI showing the method of assessing the Insall–Salvati index, calculated as the ratio of the patellar tendon length at its inner aspect (white dashed line) to the greatest diagonal length of the patella (white line). **b** Patellar alta evaluation using the Caton–Deschamps index, which is the ratio between a line measured between the inferior margin of the patellar articular surface and the anterior aspect of the tibial plateau (black line) and the greatest length of the patellar articular surface (white line)

Patella alta is related to a long patellar tendon and is considered a major factor associated with reduced contact area at the patellofemoral joint and a major contributor to patellar instability [33]. In order for the patella to engage with the femoral trochlea, a higher degree of flexion than normal is needed. Several methods have been used to assess patella alta. A commonly used one is the Insall–Salvati ratio of patellar tendon length: patellar length. This is measured on the sagittal MRI images at the point where the patella is at its greatest length. It is a ratio between the patellar tendon length (along the inner surface of the tendon) and the diagonal patellar height [27]. A ratio of > 1.3 is considered indicative of patella alta [34] (Fig. 4). Another method is the Caton–Deschamps index. This is the ratio between a line measured between the inferior margin of the patellar articular surface and the anterior aspect of the tibial plateau and the greatest length of the patellar articular surface. A ratio equal or more than 1.2 indicates patella alta [35] (Fig. 4). A newer method to assess for patella alta is the patellotrochlear index (PTI), which is measured in the midsagital MRI as the ratio of the length of trochlear cartilage engaged with the patella to the patellar cartilage length [36]. PTI of less than 12.5% suggests the presence of patella alta. Each of the mentioned assessment methods of patella alta has its own advantages and limitations. As an example, although the Insall–Salvati ratio is one of the most commonly used methods and does not depend on the degree of knee flexion, it is affected by the patellar shape particularly its inferior point and measurement does not change after tibial tubercle distalization procedure [25]. On the other hand, the PTI is significantly altered with knee flexion [37].

### Tibial tubercle–trochlear groove distance

An increased tibial tubercle–trochlear groove (TT-TG) indicates a lateralized tibial tuberosity, or a medialized trochlear groove [38]. TT-TG is a reflection of the clinically measured Q angle. A high Q angle or TT-TG would exert a lateral pressure on the patella during knee extension, and if this is not counteracted by vastus medialis muscle contraction, it can predispose to lateral patellar subluxation and instability [39, 40]. There is a degree of variability in the literature about what is considered an abnormally high TT-TG. TT-TG distance of more than 20 mm is believed to be nearly always associated with patellar instability [27].

**Fig. 5:**
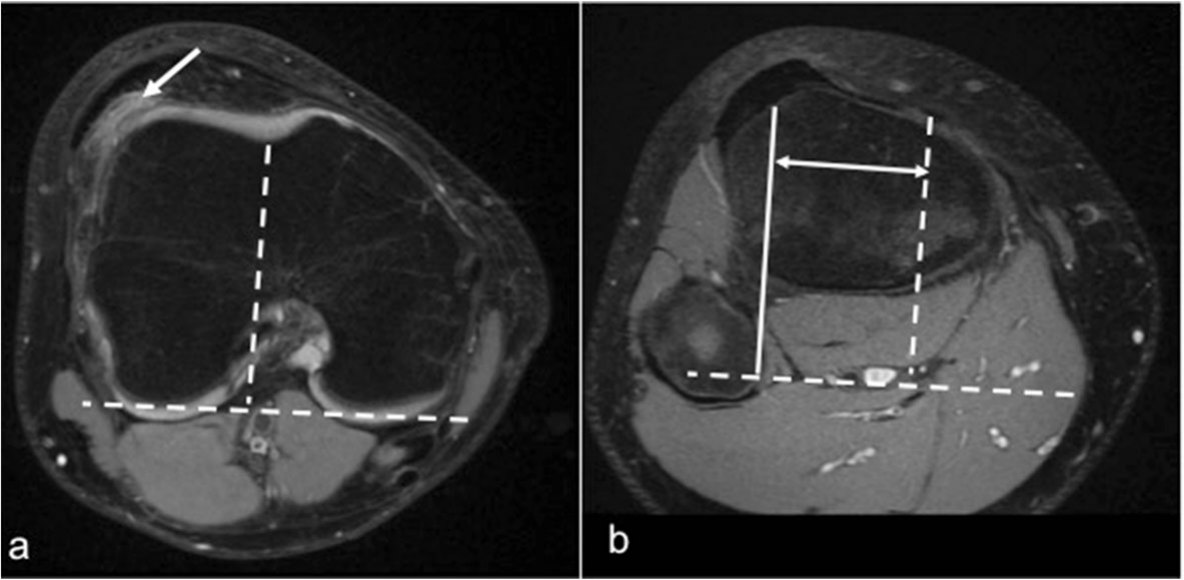
Tibial tubercle–trochlear groove distance (TT-TG) assessment. **a** Axial PDFS right knee MR image at the level of the trochlear groove. **b** Axial MRI at the level of the tibial tuberosity. TTTG is the distance between the solid and the dashed lines in (**b**). Note the edema in the superolateral aspect of Hoffa’s fat pad (arrow)

The TT-TG is evaluated by measuring the distance between the most anterior point of the tibial tuberosity and the deepest point of the trochlear groove using two lines drawn perpendicular to the tangent to the posterior borders of the femoral condyles [31] (Fig. 5). TT-TG assessment has its own limitations. The TT-TG distance can be influenced by the degree of knee flexion (reduces with flexion), and it is also smaller upon weight bearing [41]. It can be difficult to determine the deepest part of the trochlear groove when assessing the TT-TTG in the presence of trochlear dysplasia; therefore, an alternative method for assessing tibial tubercle position was proposed measuring the distance in reference to the posterior cruciate ligament and not to the trochlea (tibial tubercle-posterior cruciate ligament distance [TT-PCL]), with proposed pathologic threshold of 21 mm [42, 43]. More recently, the TT-TG index was developed, which takes knee size into account by assessing the proximal–distal distance between the entrance of the chondral trochlear groove (TE) and the tibial tuberosity (TT). The TT-TG index is the TTTG/TT-TE ratio [44].

### Lateral patellar tilt

**Fig. 6:**
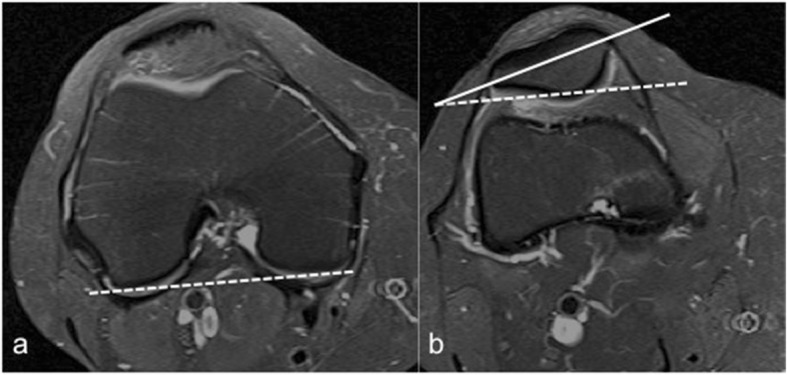
Patellar tilt assessment. **a** The posterior condylar line is drawn on the slice where the posterior femoral condyles are largest (dashed line). **b** The patella tilt angle is measured between the posterior condylar line (dashed line) and the maximal patella width (solid line).

Lateral patellar tilt is a sensitive marker for patellar instability [45]. It may occur without patellar lateralization. In a series of 474 patients with anterior knee pain, patellar tilt or subluxation was present in 40% of the cases on axial MRI [46]. The degree of patellar tilt can be evaluated by measuring the patella tilt angle, which is the angle between the posterior condylar line and the maximal patella width line [47] (Fig. 6). Patellar tilt can also be assessed using the patellofemoral angle (PFA). PFA is the angle between a line drawn along the bony lateral patellar facet and another line along the anterior aspect of the femoral condyles. It is measured at the mid-point of the patella on the axial slices [48]. PFA of 0° or if it opens medially (negative value) is considered abnormal indicating lateral patellar tilt [27, 48].

### Structural changes associated with patellar maltracking

#### Injuries to the medial patellar stabilizers

**Fig. 7:**
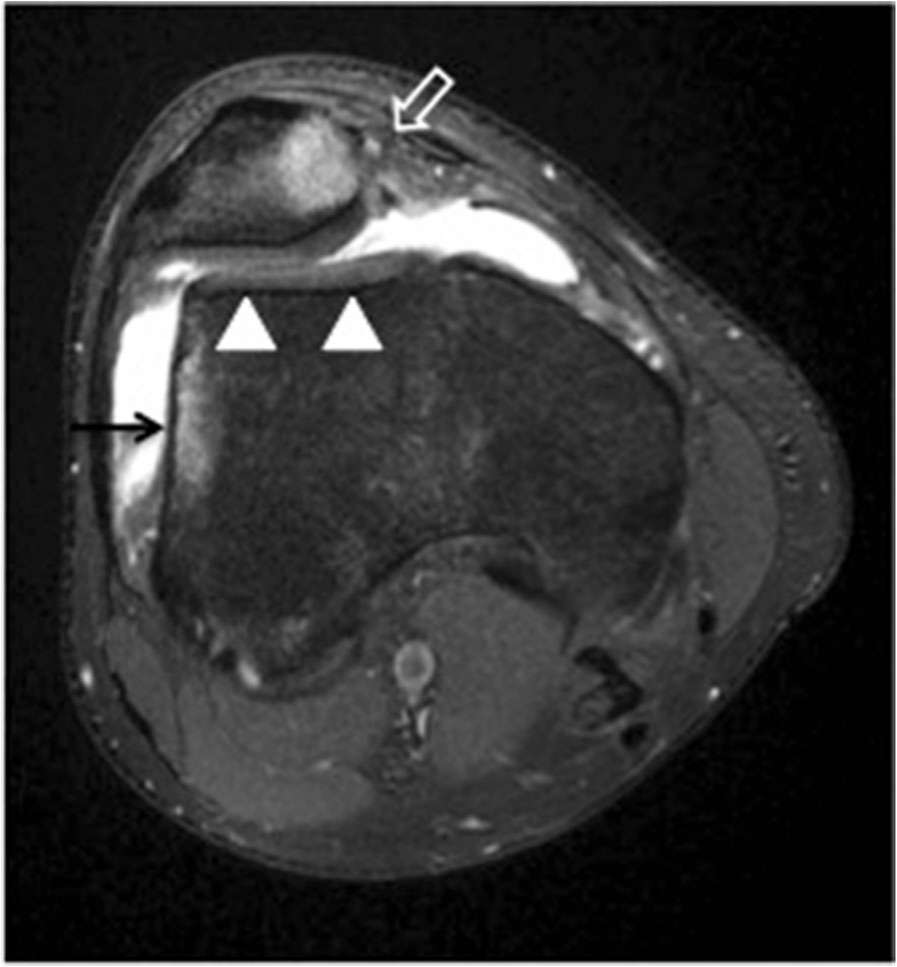
Transient lateral patellar dislocation. Axial PDFS MR image showing MPFL disruption (open arrow) and trochlear dysplasia (arrowheads). There is edema of the medial patella and of the lateral femoral condyle (arrow), consistent with bone contusion due to recent lateral patellar dislocation

It has been shown that damage to the medial patellar stabilizers including medial patellar retinaculum and the medial patellofemoral ligament (MPFL) injuries are prevalent in 70–100% of cases of lateral patellar dislocation [5, 27, 49–51]. The medial patellar retinaculum and MPFL are best seen on MRI on the axial fluid-sensitive sequences. These two structures blend with each other and are difficult to separate on imaging. The MPFL is attached to the region of the adductor tubercle or medial femoral epicondyle extending deep to the vastus medialis obliquus (VMO) and attaching to the superior two thirds of the patella [52]. In one MRI study, 76% of cases of prior lateral patellar dislocation showed medial retinacular injury at its patellar insertion and 30% at its midsubstance, and injury of the femoral origin of the MPFL was identified in 49% of the cases [49]. Both MRI and ultrasound are accurate imaging modalities in the detection of MPFL injuries [5, 50, 51]. The patellar retinaculum and the MPFL are seen on MRI as well-defined low-signal-intensity bands. The MPFL is best seen on axial MRI on the slice just distal to the VMO. On T2-weighted MR images, sprain is depicted as thickening of retinaculum with signal intensity signifying edema and hemorrhage (Fig. 7). Complete disruption and avulsion are seen as discontinuity of ligament fibers with associated edema [50]. It has been shown that ossification in the medial patellar stabilizers correlates with prior injury to these structures [53].

#### Superolateral Hoffa’s fat pad impingement

Infrapatellar (Hoffa’s) fat pad impingement is recognized as a cause of anterior knee pain. Chronic fat impingement can result in chronic inflammation and fat pad hypertrophy. On MRI, impingement is usually manifested as high signal intensity within the superolateral aspect of the infrapatellar fat pad on fluid-sensitive sequences (edema) (Fig. 5). It has been shown that there is an association between edema at the superolateral aspect of Hoffa’s fat pad and a number of patellar maltracking parameters [30, 54, 55]. It has been suggested that fat impingement occurs between the lateral femoral condyle and the posterior aspect of the patellar tendon [54]. Identifying edema at the superolateral aspect of Hoffa’s fat pad on MRI should prompt the reporting radiologist to look for features of patellar maltracking. Although edema can be seen in other peripatellar fad pads on MRI, there is no clear association between patellar maltracking and prefemoral fat pad edema or with that at the suprapatellar fat pad [56].

#### Chondral and osteochondral injuries

An imbalance of forces acting on the patellofemoral joint due to abnormal bony geometry or altered function of the active and passive soft tissue restraints may result in abnormalities of alignment and tracking of the patella. Stress and shear forces that follow can result in cartilage damage and the development and evolution of osteoarthritis [57].

MRI, given its superior soft tissue contrast and multi-planar capability, has emerged as the modality of choice in evaluating articular cartilage abnormalities. It has proven to be both sensitive and specific in the detection of hyaline cartilage abnormalities [4].

An association has been demonstrated between patellofemoral cartilage damage and patellar maltracking. A study has found that the femoral groove tends to be shallower in osteoarthritis patients compared to those with normal cartilage, regardless of age. Significantly greater lateral patellar displacement and tilt was found in osteoarthritis patients compared to a control group [3]. Another study noted an association between abnormal trochlear morphology and high-grade patellofemoral cartilage damage [58]. It was shown that certain features of patellar maltracking (increased sulcus angle, lateral patellar tilt, and a higher patellar tendon to patellar length ratio) are associated with cartilage loss and bone marrow lesions [59].

On the other hand, frank patellar dislocation is a significant risk factor in the development of patellofemoral osteoarthritis with an incidence of 49% at 25 years after the patellar dislocation incident in comparison with 8% in a control group without a dislocation history [60]. Lateral patellar dislocation results in bone contusion at the medial patella and along the lateral aspect of the lateral femoral condyle. The patellar usually relocates, and the typical bone contusions are the key MRI features to diagnose transient lateral patellar dislocation (Fig. 7). Osteochondral fractures are common in acute or recurrent transient lateral patellar dislocation, seen in up to 70% of cases. These are most often found at the inferomedial patella or the lateral femoral condyle [49, 61, 62].

### CT role on evaluating patellar maltracking

**Fig. 8:**
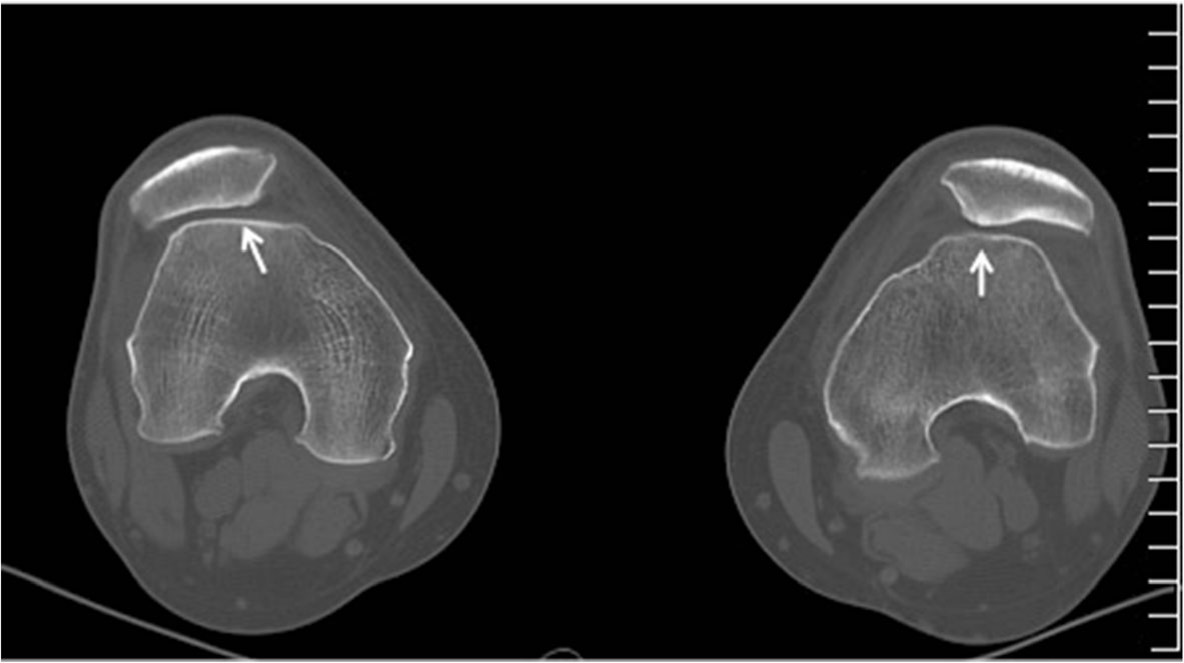
CT of both knees in 20° flexion demonstrating bilateral shallow trochlear groove (arrows) compatible with dysplasia and bilateral lateral patellar subluxation and lateral tilt. The two features are associated with patellar maltracking

For CT evaluation of the patellofemoral joint, patients are positioned supine, with mild external rotation of up to 15° with padding as needed to facilitate a relaxed state of the quadriceps musculature. Both knees are scanned simultaneously. The contralateral side may serve as an internal control or may also have anatomic factors predisposing to maltracking. This protocol can help in evaluating for osseous integrity, morphology, and patellofemoral alignment [63] (Fig. 8). However, patellofemoral tracking is a dynamic process with the spatial relationship between the articular surfaces varying depending on the position of the knee joint [57, 64]. In fact, most patellar maltracking occurs between extension and the first 30° of flexion. Thus, to assess for maltracking specifically, a multi-stage CT with a variable number of repeated acquisitions at variable degrees of flexion can also be performed [57]. At 0° extension, the patellar may lie completely above the level of the trochlea, without direct apposition between the two articular surfaces. However, the patella starts to engage with the trochlea by 30° and is typically completely engaged by 45°. At less than 30° of flexion, asymptomatic knees may demonstrate physiologic patellar tilt or subluxation. In addition, symptomatic knees may demonstrate normal engagement between the patella and trochlea beyond 30° of flexion. Thus, imaging at positions both less than and greater than 30° can be used to avoid missing maltracking that might be captured at only certain degrees of flexion [64].

Advantages of CT over plain radiography include its cross-sectional capability and ability to generate multiplanar reformations. This allows for greater detailed evaluation of the patellar and trochlear morphology, patellofemoral relationship, and status of the joint. Advantages of CT over MRI include the reduced cost, larger gantry diameter allowing to fit larger patients, faster acquisition with less potential for claustrophobia, fewer absolute and relative contraindications related to implanted devices, and better cortical bone definition. It is therefore helpful in surgical planning. Disadvantages of CT compared to MRI include the use of ionizing radiation, which reduced soft tissue contrast resulting in limited evaluation of the cartilage, tendons, ligaments, muscles, and internal structures of the knee [64].

Features that may predispose to patellar dislocation and/or patellar maltracking and can be evaluated with CT include patellar and trochlear morphology and the alignment between the two structures. These morphological risk factors can be assessed using methods similar to those on MRI as detailed in the prior sections of this article. In acute patellar dislocation, CT may demonstrate osseous impaction or fractures of the medial margin of the patella (with or without involvement of the articular surface) and/or the lateral surface of the lateral femoral condyle and intraarticular fragments. Soft tissue changes may include effusion, thickening or disruption of the MPFL, and retinacular complex and regional edema.

### Synopsis of treatment strategies

The goal of patellar instability treatment is to achieve a stable, functional, and pain-free knee and ultimately to halt or slow the development of osteoarthritis. It can be divided into nonoperative and operative management. The literature in this field has been extremely heterogeneous, and this has made clinical guidelines difficult to produce. A 2015 Cochrane Review concluded that there is no significant increase in functional scores between nonoperative and operative management; however, surgical management does result in a significantly lower risk of recurrent dislocation at the cost of surgical complications [19]. Therefore, the management of patellar maltracking remains controversial and decisions need to be made on an individual patient basis with surgical management being reserved for those patients with documented recurrent lateral patellar instability.

#### Non-operative management

For first-time dislocators without intra-articular loose bodies or chondral injury, a trial of nonoperative therapy is indicated. This treatment generally consists of the use of anti-inflammatory medications, a short period of immobilization (3–6 weeks) followed by a progressive physiotherapy regimen with focus on range of motion, closed chain exercises, and vastus medialis obliquus strengthening [16, 65–70]. Adjunctive treatments such as knee aspiration and patellar stabilizing braces have been proposed to decrease swelling and enable early range of motion; however, there is no conclusive evidence for their use [71].

Despite non-operative management, recurrent patellar instability occurs in between 15 and 45% of patients [17–20]. Additionally, return to sport can be as low as 45%, leaving many patients searching for further management options [12].

#### Surgical management

Surgical management of patellar instability should be guided on an individual patient basis depending on history, physical examination, and radiologic findings as outlined above. Over 100 different procedures have been described for the treatment of patellar instability, and this reflects the various causes for instability and lack of current gold standard [66, 69, 72]. The most accepted indication for surgical management of patellar instability is the presence of a large displaced osteochondral fracture or loose body.

Surgical management procedures can broadly be categorized as soft tissue procedures (lateral release, medial imbrication, and MPFL repair or reconstruction) and bony procedures (tibial tubercle transfer procedures and trochleoplasty).

**Fig. 9:**
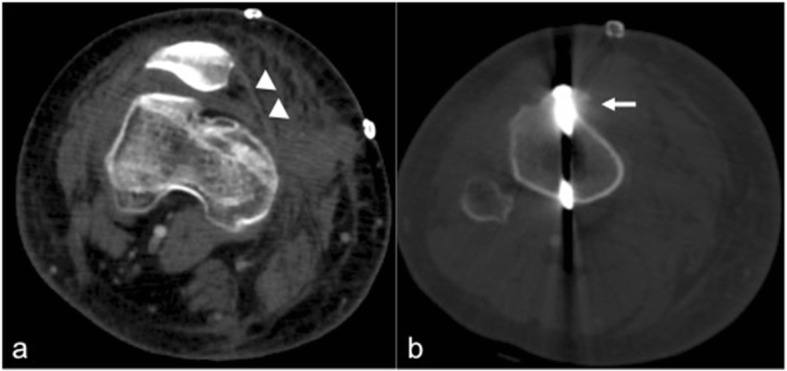
Knee CT images in the early post-operative period in a 19-year-old male with history of patellar maltracking. **a** Axial CT image demonstrating MPFL reconstruction (arrow heads). **b** Axial CT image showing tibial tuberosity transfer surgery with screw placement (arrow)

Soft tissue procedures are designed to repair or tighten stretched and injured soft tissues contributing to patellar stability. They are best indicated in isolation in the setting of recurrent instability with minimal underlying osseous malalignment (normal TT-TG, minimal trochlear dysplasia). Lateral release and medial imbrication on their own are generally insufficient, but can be used to augment an MPFL repair or reconstruction or, if there is osseous misalignment, used in conjunction with a bony procedure particularly if there is recurrent instability or demonstrable lateral patellar tilt [73–78]. Reconstruction of the MPFL has recently become an increasingly popular procedure for recurrent lateral patellar instability. The technique has been refined, and a better understanding of the anatomical features of both the origin and insertion of the ligament onto the patella has made the operation more reproducible (Fig. 9). Despite this, there remains considerable variation in surgical technique including graft choice, position, and tension making the literature difficult to compare [8, 15, 79–86].

In the setting of osseous patellar malalignment, an osseous procedure such as tibial tubercle transfer osteotomy can be performed (Fig. 9). Several osteotomies have been described including the medializing Elmslie–Trillat procedure, the anteromedializing Fulkerson osteotomy, and distalization osteotomy [87]. By altering the insertion point of the patellar tendon, these procedures affect the timing and position of patellar engagement in the trochlea and have the ability to biomechanically offload damaged distal articular cartilage, thereby reducing pain and increasing stability simultaneously. It should be noted that these procedures are mostly contraindicated in the patient with open physes due to growth arrest of the tibial tubercle apophysis.

Lastly, a sulcus-deepening procedure known as trochleoplasty may be indicated in the patient with significant trochlear dysplasia and recurrent instability. This procedure involves removal of cancellous bone beneath the trochlea followed by fixation of the articular surface [88, 89].

## Conclusion

Patellar maltracking is a disorder that often affects the young active individuals. Early detection particularly in the stage preceding the development of significant cartilaginous loss and osteoarthritis is critical. Imaging plays a vital role in detecting not only the secondary damage but also subtle early features that can raise the suspicion for the presence of this entity. This can provide a road map of developing a treatment strategy that would be primarily aimed at stabilizing the patellofemoral joint and halt the progression of cartilage loss.

## Data Availability

Not applicable

## Abbreviations

AP: Anteroposterior
CT: Computed tomography
MPFL: Medial patellofemoral ligament
MRI: Magnetic resonance imaging
TT-TG: Tibial tubercle–trochlear groove
